# Methanol-based cadaverine production by genetically engineered Bacillus methanolicus strains

**DOI:** 10.1111/1751-7915.12257

**Published:** 2015-01-23

**Authors:** Ingemar Nærdal, Johannes Pfeifenschneider, Trygve Brautaset, Volker F Wendisch

**Affiliations:** 1 Sector for Biotechnology and Nanomedicine, Department of Molecular Biology, SINTEF Materials and ChemistryTrondheim, Norway; 2 Genetics of Prokaryotes, Faculty of Biology & CeBiTec, Bielefeld UniversityBielefeld, Germany; 3 Department of Biotechnology, Norwegian University of Science and TechnologyTrondheim, Norway

## Abstract

Methanol is regarded as an attractive substrate for biotechnological production of value-added bulk products, such as amino acids and polyamines. In the present study, the methylotrophic and thermophilic bacterium *Bacillus methanolicus* was engineered into a microbial cell factory for the production of the platform chemical 1,5-diaminopentane (cadaverine) from methanol. This was achieved by the heterologous expression of the *Escherichia coli* genes *cadA* and *ldcC* encoding two different lysine decarboxylase enzymes, and by increasing the overall L-lysine production levels in this host. Both CadA and LdcC were functional in *B. methanolicus* cultivated at 50°C and expression of *cadA* resulted in cadaverine production levels up to 500 mg l^−1^ during shake flask conditions. A volume-corrected concentration of 11.3 g l^−1^ of cadaverine was obtained by high-cell density fed-batch methanol fermentation. Our results demonstrated that efficient conversion of L-lysine into cadaverine presumably has severe effects on feedback regulation of the L-lysine biosynthetic pathway in *B. methanolicus*. By also investigating the cadaverine tolerance level, *B. methanolicus* proved to be an exciting alternative host and comparable to the well-known bacterial hosts *E. coli* and *Corynebacterium glutamicum*. This study represents the first demonstration of microbial production of cadaverine from methanol.

## Introduction

**Figure 1:**
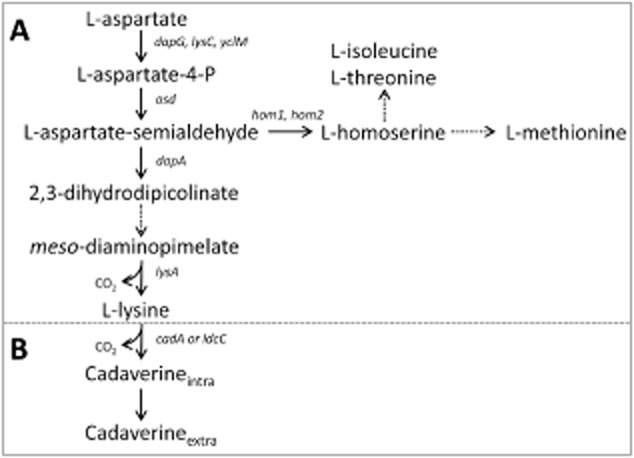
Pathway for L-lysine and cadaverine biosynthesis in *B. methanolicus.* Gene names are indicated next to arrows representing reactions. Dotted arrows summarize several reactions. Reactions present in box A are endogenous in *B. methanolicus*, whereas reactions in box B involve the decarboxylation of L-lysine due to the heterologous expression of E. coli genes and export mechanisms of cadaverine to the extracellular medium.

There is a high societal demand for – and scientific interest in – more environmental-friendly and sustainable production processes for large quantity bulk products. As examples, amino acids and polyamines find applications as food/feed additives as well as in the pharmaceutical, plastics and polymer industry (Wendisch, 2014). The polyamine monomer 1,5-diaminopentane, commonly known as cadaverine, is a sought-after platform chemical used for production of various polyamides and is currently mainly fabricated by petroleum-based chemical synthesis. With the increasing focus on bio-economy and low-carbon footprints in the industry, efforts have been made to develop biotechnological production processes for several polyamines (Adkins *et al*., 2012; Buschke *et al*., 2013; Meiswinkel *et al*., 2013a). Applying bacteria as microbial production hosts, certain polyamines can be obtained from amino acids including L-lysine, L-arginine and L-ornithine by thermodynamically favourable decarboxylation reactions (Schneider and Wendisch, 2011). These amino acids can be obtained by microbial fermentation processes and the worldwide production of the feed amino acid L-lysine amounts to almost 2 million tons per year (Wendisch, 2014). The common approach has been to establish L-lysine overproducing hosts for the concomitant engineering towards efficient production of cadaverine, as this compound is formed by a one-step conversion of L-lysine catalysed by lysine decarboxylase (Kind *et al*., 2010; Kind and Wittmann, 2011; Qian *et al*., 2011) (Fig. 1). In particular, the genes of the lysine decarboxylases found naturally in *Escherichia coli*, encoded by *cadA* and *ldcC*, have been applied and overexpressed. Also cadaverine secretion has been a target for optimization of production (Kind *et al*., 2011; Li *et al*., 2014). Typically, these production processes rely on *E. coli* and *Corynebacterium glutamicum* as microbial hosts using sugars from molasses or from starch hydrolysis as carbon and energy substrates leading to an unwanted competition with human food supply, and consequently nutrition prices are rising worldwide (Schrader *et al*., 2009). As an alternative, e.g. recombinant *C. glutamicum* strains have been developed to accept alternative carbon sources such as glycerol from the biodiesel process (Meiswinkel *et al*., 2013a), amino sugars derived from chitin (Uhde *et al*., 2013; Matano *et al*., 2014) and pentoses present in lignocellulosic hydrolysates (Gopinath *et al*., 2011; Meiswinkel *et al*., 2013b). More generally, the possibility to produce polyamines, amino acids and other bulk products and biofuels from alternative non-food carbon sources has been in the research focus of biotechnology in recent years. The one-carbon substrate methanol has long been regarded as a convenient fuel and raw material for manmade hydrocarbon-based products (Olah, 2005). It occurs abundantly throughout nature, it is a pure raw material that can be completely utilized in microbial fermentation processes, and the price is expected to remain independent from and lower than sugar prices (Brautaset *et al*., 2007; Schrader *et al*., 2009). Based on all this, methanol is regarded as a highly attractive non-food substrate for microbial bioprocesses.

The Gram-positive and facultative methylotrophic bacterium *Bacillus methanolicus* is able to utilize methanol as sole carbon and energy source for growth (Müller *et al*., 2014). As methanol growth is characterized by high oxygen demands leading to an increased heat output, it is an advantage that *B. methanolicus* has a growth optimum at 50–55°C, reducing the process cooling costs. The genome sequences of two wild-type *B. methanolicus* strains MGA3 and PB1 were recently published (Heggeset *et al*., 2012; Irla *et al*., 2014a) and its transcriptome characterized (Irla *et al*., 2014b) serving as a solid basis for increased understanding of methylotrophy and product formation, e.g. L-glutamate and L-lysine, in this industrially relevant bacterium. It has been well documented that *B. methanolicus* has great potential for L-lysine overproduction through classical mutagenesis studies and selection of strains resistant to the L-lysine analog S-2-aminoethylcysteine (Hanson *et al*., 1996; Brautaset *et al*., 2010). Several key genes and enzymes of the aspartate pathway of *B. methanolicus* have been characterized, and insight into genetic repression and feedback inhibition has been established (Jakobsen *et al*., 2009; Brautaset *et al*., 2010). Furthermore, metabolic engineering of central metabolism and the aspartate pathway towards L-lysine in the MGA3 wild-type strain yielded significant L-lysine overproduction during shake flask experiments and fed-batch fermentations (Nærdal *et al*., 2011). The theoretical maximum L-lysine yield from methanol has been calculated to 0.82 g g^−1^ in this bacterium (Brautaset *et al*., 2007), which is comparable to the estimated maximum L-lysine yield from glucose in *C. glutamicum* (de Graaf, 2000; Wittmann and Becker, 2007). Thus, *B. methanolicus* was regarded as a potential promising host for production of cadaverine from methanol.

In the present study, we have investigated the potential of methanol-based biotechnological production of cadaverine at elevated temperature using wild-type and mutant *B. methanolicus* strains as hosts. Since inspection of the genome sequence did not reveal a gene putatively encoding a lysine decarboxylase (Fig. 1), synthetic cadaverine production modules based on the lysine decarboxylase isozymes LdcC and CadA from Gram-negative *E. coli* were constructed and heterologously expressed in *B. methanolicus* strains. Both enzymes proved functional and resulted in cadaverine production in *B. methanolicus*, and CadA overexpression provided the highest cadaverine production levels. This is to our knowledge the first demonstration of microbial cadaverine production from methanol.

## Results

### Bacillus methanolicus lacks cadaverine biosynthetic and degradation genes and tolerates up to 200 mM cadaverine before growth is severely affected

The genome sequencing of the wild-type *B. methanolicus* strains MGA3 and PB1 (Heggeset *et al*., 2012; Irla *et al*., 2014a) has identified all genes of the aspartate pathway leading to L-lysine, while genes putatively encoding L-lysine decarboxylases for conversion of L-lysine to cadaverine were not found. Furthermore, no putative cadaverine exporter genes were identified in the *B. methanolicus* genomes. Hence, heterologous expression of a lysine decarboxylase gene in *B. methanolicus* is a requirement for cadaverine production which has never been reported for this species.

To test the tolerance of *B. methanolicus* to cadaverine, this compound was added to exponentially growing cells and growth was monitored. For this purpose, the *B. methanolicus* strain M168-20 was used and cultivated in shake flasks containing methanol (MeOH_200_) medium. The cells were grown to an OD_600_ of 0.4 before different concentrations of cadaverine dihydrochloride (0–200 mM, corresponding to 0–35 g l^−1^) were added to triplicate cultures for each concentration. The control cultures without cadaverine supplementation grew with a specific growth rate (μ) of 0.46 ± 0.01 h^−1^ to an OD_600_ of 8.7 ± 0.14. With the addition of 50 mM, 100 mM and 200 mM of cadaverine dihydrochloride, the maximum OD_600_ values obtained were 7.5 ± 0.18, 6.2 ± 0.20 and 5.5 ± 0.22, respectively, and the accompanied specific growth rates were also reduced (0.40 ± 0.02 h^−1^, 0.39 ± 0.01 h^−1^ and 0.36 ± 0.01 h^−1^). Thus, a minor growth inhibition by cadaverine was observed since addition of 200 mM (35 g l^−1^) cadaverine dihydrochloride reduced the growth rate by about 20%.

Cadaverine may be degraded by certain bacteria and the involved genes have been identified (Schneider and Wendisch, 2011). However, inspection of the *B. methanolicus* MGA3 and PB1 genomes did not indicate that this bacterium is capable of catabolizing cadaverine. This was also experimentally confirmed in shake flask cultures by substituting methanol and ammonium sulphate, as carbon and nitrogen source, respectively, with cadaverine. Cadaverine did not support bacterial growth, and the cadaverine concentration did not decrease throughout the cultivation as analysed by reverse-phase high-performance liquid chromatography (data not shown).

### Heterologous expression of L-lysine decarboxylase genes enabled methanol-based cadaverine production by B. methanolicus classical mutant strain M168-20

Since *B. methanolicus* lacks a lysine decarboxylase gene, the lysine decarboxylase genes *ldcC* and *cadA* from *E. coli* MG1655 were cloned into a pHP13 derivative expression vector carrying the strong *mdh* promoter for overexpression and used to transform the L-lysine overproducing classical mutant *B. methanolicus* strain M168-20. To assay for functional expression of *ldcC* and *cadA*, respectively, crude extracts of strains M168-20(pTH1mp-*ldcC*) and M168-20(pTH1mp-*cadA*) were prepared, and the specific L-lysine decarboxylase activities were determined (Table 1). The protein concentrations of the crude extracts of M168-20(pHP13), M168-20(pTH1mp-*ldcC*), and M168-20(pTH1mp-*cadA*) were 7.0 ± 0.3 mg, 7.8 ± 0.5 mg and 12.0 ± 1.5 mg respectively. L-lysine decarboxylase activity could not be detected in the empty vector control (< 1 nmol min^−1^ mg^−1^), whereas expression of *ldcC* and of *cadA* resulted in L-lysine decarboxylase activities of 7 ± 1 nmol min^−1^ mg^−1^ in M168-20(pTH1mp-*ldcC*) and of 88 ± 11 nmol min^−1^ mg^−1^ in M168-20(pTH1mp-*cadA*) (Table 1).

**Table 1:** Specific L-lysine decarboxylase activities, cadaverine and L-lysine production levels in recombinant *B. methanolicus* M168-20 strains

Plasmid	L-lysine decarboxylase specific activity	Cadaverine	L-lysine	Cadaverine + L-lysine
	nmol/min/mg protein	mg/L	mg/L	mg/L
pHP13	< 1 ± 0.2	0	140 ± 10	140
pTH1mp-*ldcC*	7.0 ± 1.0	130 ± 10	40 ± 5	170
pTH1mp-*cadA*	88.0 ± 11.0	420 ± 25	10 ± 2	430

The results shown are from triplicate (cadaverine and L-lysine) and duplicate (lysine decarboxylase activity) shake flask cultures. Activity was measured using crude extracts from exponentially growing cells, whereas the production levels were found from late stationary cultures, approximately 20 h after inoculation.

Subsequently, production experiments were carried out with *B. methanolicus* strains M168-20(pTH1mp-*ldcC*) and M168-20(pTH1mp-*cadA*) at 50°C in 500 ml shake flask cultures with MeOH_200_ medium pH 7.2, and samples were harvested and analysed by HPLC, as described in *Experimental procedures*. As experimental control, the M168-20 strain transformed with the empty vector pHP13 was included. In accordance with previously reported data, the M168-20 (pHP13) strain produced 140 ± 10 mg l^−1^ of L-lysine under these conditions (Nærdal *et al*., 2011) and, as expected, no cadaverine production was detected. The heterologous expression of *ldcC* in *B. methanolicus* M168-20 resulted in production of 130 ± 10 mg l^−1^ cadaverine and a L-lysine level of 40 ± 5 mg l^−1^ (Table 1), confirming that the *ldcC* encoded lysine decarboxylase functions *in vivo* in *B. methanolicus* at 50°C. Similarly, heterologous expression of *cadA* entailed a surprisingly high cadaverine production level of 420 ± 25 mg l^−1^ and only 10 ± 2 mg l^−1^ L-lysine could be detected as side product (Table 1). Thus, methanol-based production of cadaverine by *B. methanolicus* was achieved. Notably, combined formation of cadaverine and L-lysine by the *cadA* and *ldcC* expressing strains was above threefold higher than L-lysine formation by the parent strain (Table 1), which might indicate feedback deregulation by L-lysine as consequence of a metabolic pull by lysine decarboxylase.

### Effect of the medium pH on cadaverine production by recombinant B. methanolicus

Since LdcC and CadA function in pH homeostasis in *E. coli*, the effect of varying the pH of the production media on cadaverine production was investigated. *B methanolicus* strains M168-20(pHP13), M168-20(pTH1mp-*ldcC*) and M168-20(pTH1mp-*cadA*) were cultivated in MeOH_200_ medium adjusted to different pH values ranging from pH 6.5 to 8.5 prior to autoclaving. The standard MeOH_200_ medium pH of 7.2 was included as control in these shake flask experiments for direct comparison. The control strain M168-20(pHP13) was included to test for any potential pH effects on L-lysine production. L-lysine production by M168-20(pHP13) was reduced to about 50 ± 5 mg l^−1^ at slightly acidic pH (pH 6.5), but remained stable (130–140 mg l^−1^) at slightly alkaline pH (pH 7.2 to 8.5). Cadaverine production by M168-20(pTH1mp-*ldcC*) was lower at pH 6.5 (52 ± 5 mg l^−1^) than at pH 7.2 (135 ± 10 mg l^−1^), but about twofold higher at pH values between 7.6 and 8.5 (about 300 mg l^−1^; Table 2). However, the productivity was maximal at pH 7.6 since the growth rate decreased at higher pH values (data not shown). Strain M168-20(pTH1mp-*cadA*) accumulated similar concentrations of cadaverine (430 to 520 mg l^−1^) at all tested pH values except at pH 6.5 (45 ± 5 mg l^−1^), a condition also characterized by reduced production of the immediate precursor L-lysine (Table 3).

**Table 2:** Production of cadaverine and L-lysine by recombinant *B. methanolicus* M168-20 strains cultivated at different medium pH

pH	M168-20(pHP13)		M168-20(pTH1mp-*ldcC*)		M168-20(pTH1mp-*cadA*)	
	Cadaverine	L-lysine	Cadaverine	L-lysine	Cadaverine	L-lysine
6.5	0	50 ± 10	52 ± 5	< 15	45 ± 5	< 15
7.2	0	130 ± 10	135 ± 10	40 ± 5	430 ± 20	< 30
7.6	0	140 ± 10	315 ± 20	< 30	450 ± 20	< 30
8.0	0	140 ± 10	305 ± 30	< 15	500 ± 30	< 30
8.5	0	140 ± 10	305 ± 30	< 15	520 ± 30	< 30

The mean values (mg/L) and standard deviation of triplicate shake flask cultures is presented. The production levels were found from late stationary cultures, from 20–30 h after inoculation.

**Table 3:** Cadaverine and L-lysine production by recombinant *B. methanolicus* MGA3 strains

Plasmid	Cadaverine	L-lysine
	mg/L	mg/L
pHP13	0	7 ± 1^a^
pTH1mp-*lysC*	0	55 ± 5^a^
pTH1mp-*lysA*	0	150 ± 10^a^
pTH1mp-*ldcC*	20 ± 4	7 ± 1
pTH1mp-*ldcC*-*lysC*	140 ± 10	< 10
pTH1mp-*ldcC*-*lysA*	190 ± 10	< 10
pTH1mp-*cadA*	450 ± 30	< 10
pTH1mp-*cadA*-*lysA*	480 ± 30	< 10

a Data imported from (Nærdal *et al*., 2011).

The production levels were found from late stationary shake flask cultures, approximately 20 h after inoculation.

As lysine decarboxylase activity is reported to depend on pyridoxal-5-phosphate (PLP) as cofactor, addition of pyridoxal-5-phosphate hydrate (1 mg l^−1^) to MeOH_200_ medium at pH 7.6 was tested. However, PLP supply in *B. methanolicus* was not limiting cadaverine production under the chosen conditions since production did not increase upon addition of pyridoxal phosphate (data not shown).

### Construction of cadaverine overproducing strains by using the wild-type B. methanolicus MGA3 as a host

We have previously achieved L-lysine overproduction by engineering of the aspartate pathway and using wild-type *B. methanolicus* strain MGA3 as host. For example, overexpression of the genes *lysC* and *lysA*, encoding aspartokinase II and meso-diaminopimelate decarboxylase, respectively, resulted in L-lysine overproduction (Nærdal *et al*., 2011). We hypothesized that coupled overexpression of these two genes together with the *ldcC* and *cadA* genes in MGA3 could result in effective cadaverine production. The recombinant strains MGA3(pTH1mp-*ldcC*-*lysC*), MGA3(pTH1mp-*ldcC*-*lysA*) and MGA3(pTH1mp-*cadA*-*lysA*) were therefore constructed. To investigate if heterologous expression of *ldcC* and *cadA* alone entails cadaverine production in MGA3, strains MGA3(pTH1mp-*ldcC*) and MGA3(pTH1mp-*cadA*) were also established. Expression of *ldcC* alone resulted in only minor cadaverine production (20 ± 4 mg l^−1^), while coupled overexpression with endogenous *lysC* and *lysA* improved cadaverine production (140 ± 10 and 190 ± 10 mg l^−1^), and these strains produced 10 mg l^−1^ of L-lysine (Table 3). Interestingly, L-lysine production was in each case lower (7, 55 and 150 mg l^−1^, respectively; Table 3) for the three isogenic strains that do not express *ldcC*, i.e. MGA3(pHP13), MGA3(pTH1mp-*lysC*) and MGA3(pTH1mp-*lysA*), respectively, indicating that LdcC exerts a metabolic pull deregulating flux through the L-lysine biosynthesis pathway. This notion is supported by the finding that heterologous expression of *cadA* alone in MGA3 resulted in 450 ± 30 mg l^−1^ cadaverine production (Table 3). The coupled overexpression of *cadA* with the endogenous *lysA* gene did not significantly increase cadaverine production further as 480 ± 30 mg l^−1^ was measured.

### Fed-batch methanol cultivation of strain MGA3(pTH1mp-cadA) lead to the substantial volumetric production level of 11.3 g l^−1^

We chose to investigate the promising cadaverine production strain MGA3(pTH1mp-*cadA*) during high-cell-density fed-batch methanol fermentation conditions. This strain was tested in duplicates and samples for cadaverine and amino acid analysis, cell dry weight and OD_600_ were taken throughout the cultivation. Due to the significant increase in culture volume, all values were volume corrected by multiplying with the respective correction factor. We have previously cultivated strain MGA3(pHP13) at the same fed-batch conditions and reported volume corrected values as published in (Brautaset *et al*., 2010). From these data we know that L-glutamate accumulate throughout the cultivation (59 g l^−1^), whereas the L-lysine level remain low (0.4 g l^−1^), and no cadaverine can be detected (Table 4). As also observed in shake flask studies, cadaverine accumulated during the fed-batch cultivation, but during fed-batch conditions MGA3(pTH1mp-*cadA*) reached a high volumetric yield, i.e. a volume-corrected concentration of 11.3 g l^−1^ cadaverine (Table 4). At the same time, no L-lysine could be detected. Despite of the high cadaverine production, high levels of L-glutamate and biomass was still measured indicating that the cadaverine production did not negatively affect these parameters. However, a slight reduction of the specific growth rate was observed (Table 4). The MGA3(pTH1mp-*cadA*) production levels of L-aspartate and L-alanine were similar to previously reported values for MGA3(pHP13).

**Table 4:** Fed-batch methanol fermentation production data of strains MGA3(pTH1mp-*cadA*) and MGA3(pHP13)

Strain	CDW^b^	μ^a^	Asp^b^	Glu^b^	Ala^b^	Lys^b^	Cad^b^
	g/L	h^−1^	g/L	g/L	g/L	g/L	g/L
MGA3(pTH1mp-*cadA*)	65.5	0.45	1.5	71.8	10.2	0.0	11.3
MGA3(pHP13)	45.0	0.49	1.1	59.0	12.0	0.4	0.0

a Specific growth rates are maximum values calculated from the exponential growth period.

b CDW, cadaverine and amino acid concentrations are maximum values and volume corrected (see ‘Experimental Procedures’ section).

The maximum mean values from early stationary (CDW) or late stationary growth phase are presented for the MGA3(pTH1mp-*cadA*) duplicate cultures and the deviation never exceed ten per cent. The MGA3(pHP13) data were imported from (Brautaset *et al*., 2010).

CDW, cell dry weight; μ, specific growth rate; Asp, L-aspartate; Glu, L-glutamate; Ala, L-alanine; Lys, L-lysine, Cad, cadaverine.

## Discussion

Methanol-based cadaverine production was shown here for the first time. The tolerance level of the thermophilic methylotroph *B. methanolicus* towards the end-product cadaverine was found to be similar to that of the natural cadaverine producer *E. coli*. 200 mM cadaverine added to the growth medium resulted in reduced growth rates by *B. methanolicus* and *E. coli* by 20% and 35% respectively (Qian *et al*., 2011). Reports using agar plate assays suggested a slightly higher cadaverine tolerance of *C. glutamicum* (Mimitsuka *et al*., 2007). Due to its tolerance to cadaverine and its proven inability to degrade this compound, *B. methanolicus* appears to be a suitable host for the production of cadaverine. Heterologous expression of both *ldcC* and *cadA* resulted in cadaverine production in *B. methanolicus*. Cadaverine production level was higher with *cadA* than with *ldcC* in both *B. methanolicus* host strains MGA3 and M168-20. Production of L-lysine as significant by-product was observed in an *ldcC* expressing strain (40 mg l^−1^ by M168-20(pTH1mp-*ldcC*) at pH 7.2). The *in vitro* pH optima of LdcC and CadA are reported to be 7.6 (Yamamoto *et al*., 1997; Lemonnier and Lane, 1998) and 5.7 (Moreau, 2007) respectively. The low pH optimum of CadA fits to its role in L-lysine dependent acid stress response of *E. coli* where *cadA* expression is induced at low pH and in the presence of L-lysine by the positive regulator CadC (Kuper and Jung, 2005). The intracellular pH of *B. methanolicus* has not yet been experimentally tested. A slightly acidic pH of the cultivation medium reduced L-lysine production, and as consequence lower cadaverine production was observed (Table 2). At slightly alkaline medium pH reduced L-lysine synthesis did not limit cadaverine production. Notably, in each isogenic strain pair analysed, cadaverine production due to heterologous L-lysine decarboxylase production was higher than L-lysine production by the respective parent strain. We propose that intracellular L-lysine concentrations are low as result of LdcC or CadA activity and that key aspartate pathway enzymes are relieved from feedback inhibition by L-lysine and/or their synthesis is relieved from repression by L-lysine. Indeed, AKII and DAP decarboxylase are known to be feedback inhibited by L-lysine (Mills and Flickinger, 1993; Jakobsen *et al*., 2009).

Expression of *cadA* in *B. methanolicus* strains led to higher cadaverine production than expression of *ldcC* (Tables 1, 2 and 3). Two factors may explain this finding. First, *cadA* expression led to higher L-lysine decarboxylase activities in crude extracts as compared with *ldcC* expression (Table 1). Second, CadA is reported to display a higher affinity to L-lysine than LdcC with Km values for L-lysine of 0.84 mM and 0.27 mM respectively (Krithika *et al*., 2010).

We could demonstrate high-level cadaverine production during high-cell-density fed-batch methanol fermentation of strain MGA3(pTH1mp-*cadA*). Whereas no L-lysine accumulated during the fermentation, the volume corrected production level of cadaverine reached 11.3 g l^−1^ after 30 h and remained stable throughout the cultivation time of 47 h. The volume corrected concentrations of biomass (65.5 g l^−1^) and L-glutamate (71.8 g l^−1^) obtained for MGA3(pTH1mp-*cadA*) were slightly higher than previously reported values for MGA3(pHP13) (Table 4). The finding that cadaverine could accumulate to higher concentrations in the fermenter than in shake flasks may in part be explained by the fact that the fermenter was pH-controlled and that the shake flask cultures acidified with time (data not shown). Moreover, higher cadaverine concentrations were tolerated by *B. methanolicus* since only minor negative effects on biomass and specific growth rate were observed upon addition of up to 35 g l^−1^ (200 mM) pH-adjusted cadaverine. It was observed that the cadaverine concentration increased throughout the growth phase until the early stationary phase, as also reported previously for *E. coli* and *C. glutamicum* (Kind *et al*., 2011; Qian *et al*., 2011). Due to the significant accumulation of L-glutamate in strain MGA3(pTH1mp-*cadA*) during fed-batch fermentation, there should be a great potential to increase cadaverine production further, especially by coexpression of the 2-oxoglutarate dehydrogenase from *B. methanolicus* recently found to reduce L-glutamate production 5-fold and increase L-lysine production twofold in *B. methanolicus* M168-20 (Krog *et al*., 2013). An improved understanding of both L-lysine and cadaverine secretion in *B. methanolicus* and heterologous expression of relevant known exporter or permease genes like *cadB* from *E. coli* (Li *et al*., 2014) and *cg2893* from *C. glutamicum* (Kind *et al*., 2011) could certainly be valuable for future high-level methanol-based cadaverine production in *B. methanolicus*.

## Experimental Procedures

### Biological materials, deoxyribonucleic acid manipulations and growth conditions

Bacterial strains and plasmids used in this study are listed in Table 5. *E. coli* DH5α was used as a general cloning host. *E. coli* strains were cultivated in liquid and on solid lysogeny broth medium at 37°C and standard recombinant deoxyribonucleic acid (DNA) procedures were performed as described elsewhere (Sambrook *et al*., 2011). *B. methanolicus* strains were cultivated at 50°C and 200 r.p.m. in methanol (MeOH_200_) medium (Jakobsen *et al*., 2006) containing salt buffer (4.1 g l^−1^ K_2_HPO4, 1.3 g l^−1^ NaH_2_PO_4_, 2.1 g l^−1^ (NH_4_)_2_SO_4_) and 0.025% yeast extract (Difco) adjusted to pH 7.2 unless stated otherwise. After autoclavation, the medium was supplemented with 1 mM MgSO_4_, vitamins, trace metals and 200 mM methanol as described elsewhere (Schendel *et al*., 1990; Jakobsen *et al*., 2006). The transformation of *B. methanolicus* was performed by electroporation as described previously (Jakobsen *et al*., 2006). For classical *B. methanolicus* mutant strain M168-20 (Brautaset *et al*., 2010) the growth medium was supplemented with D,L-methionine (1.5 mM). Recombinant *E. coli* and *B. methanolicus* strains were cultivated in media supplemented with chloramphenicol (15 and 5 μg ml^−1^ respectively). Bacterial growth was monitored by measuring optical density at 600 nm (OD_600_). Tolerance of *B. methanolicus* to cadaverine was investigated by monitoring bacterial growth in the presence of different cadaverine concentrations. Cadaverine dihydrochloride (Sigma Aldrich Biochemie GmbH, Hamburg, Germany) was dissolved in MeOH_200_ medium, and the solution was pH adjusted to 7.2 and pre-warmed before cadaverine was supplemented in different concentrations to the growing cell cultures. Control cultures without cadaverine were included.

**Table 5:** Bacterial strains and plasmids used in this study

Strain or plasmid	Description	Reference
*E. coli*		
DH5α	General cloning host	Stratagene
MG1655	Wild type strain	ATCC 47076
*B. methanolicus*		
MGA3	Wild-type strain	ATCC 53907
M168-20	AEC-resistant *hom1* MGA3 mutant	(Brautaset *et al*., 2010)
Plasmids		
pHP13	*E. coli*-*B. subtilis* shuttle vector, Clm^r^	(Haima *et al*., 1987)
pTH1mp-*lysC*	pHP13 derivate with *lysC* under control of *mdh* promoter	(Brautaset *et al*., 2010)
pTH1mp-*lysA*	pHP13 derivate with *lysA* under control of *mdh* promoter	(Nærdal *et al*., 2011)
pTH1mp-*ldcC*	pHP13 derivate with *ldcC* under control of *mdh* promoter	This study
pTH1mp-*cadA*	pHP13 derivate with *cadA* under control of *mdh* promoter	This study
pTH1mp-*ldcC*-*lysC*	pTH1mp-*ldcC* with *lysC* downstream of the *ldcC* gene	This study
pTH1mp-*ldcC*-*lysA*	pTH1mp-*ldcC* with *lysA* downstream of the *ldcC* gene	This study
pTH1mp-*cadA*-*lysA*	pTH1mp-*cadA* with *lysA* downstream of the *cadA* gene	This study

Clm^r^, chloramphenicol resistance.

### Construction of expression vectors

The *ldcC* gene of *E. coli* MG1655 was polymerase chain reaction (PCR) amplified from genomic DNA using primers ldcC-PciI-Fwd: 5'- GCTGCACATGTGAACATCATTGCCATTATGG-3' and ldcC-XbaI-Rev 5'-GCTGCTCTAGATTATCCCGCCATTTTTAGGAC-3'. The resulting 2162 bp PCR product was digested with *Pci*I and *Xba*I (restriction sites underlined) and ligated into corresponding sites of pTH1mp-*lysC* (replacing *lysC*) resulting in plasmid pTH1mp-*ldcC*. Vector pTH1mp-*lysC* was digested with *Spe*I and *Nco*I and the 2017 bp fragment containing *lysC* was ligated into the *Xba*I (compatible with *Spe*I) and *Nco*I sites of pTH1mp-*ldcC* resulting in plasmid pTH1mp-*ldcC*-*lysC*. Vector pTH1mp-*lysA* was digested with *Spe*I and *Nco*I, and the 1834 bp fragment containing *lysA* was ligated into the *Xba*I (compatible with *Spe*I) and *Nco*I sites of pTH1mp-*ldcC* resulting in plasmid pTH1mp-*ldcC*-*lysA*. The *cadA* gene (2148 bp) was PCR amplified from genomic DNA isolated from *E. coli* MG1655 using the following primer pair: cadA-fw: 5'-AGGAGGTAGTACATGTGAACGTTATTGCAATATTGAATC-3’ and cadA-rv: 5'-CCTATGGCGGGTACCTTATTTTTTGCTTTCTTCTTTCAA-3’. The obtained PCR product was ligated into the vector pTH1mp-*lysC*, digested with *Pci*I/*Kpn*I (replacing the *lysC* gene), using the isothermal DNA assembly method (Gibson *et al*., 2009) yielding expression vector pTH1mp-*cadA*. Vector pTH1mp-*lysA* was digested with *Spe*I and *Nco*I, and the 1834 bp fragment containing *lysA* was ligated into the *Xba*I (compatible with *Spe*I) and *Nco*I sites of pTH1mp-*cadA* resulting in plasmid pTH1mp-*cadA*-*lysA*.

### Lysine decarboxylase activity assays in B. methanolicus crude extracts

The lysine decarboxylase activity was determined in *B. methanolicus* crude cell extracts. The preparation of crude cell extracts was performed as described elsewhere previously (Brautaset *et al*., 2004). The cells were inoculated from a glycerol stock and grown in MeOH_200_ medium overnight before they were transferred to fresh MeOH_200_ medium and grown to an OD_600_ of 1.5 to 2.0. Forty millilitre of the culture was harvested by centrifugation (4000 × g, 30 min, 4°C), washed in 100 mM sodium citrate buffer (pH 7.5) and stored at –20°C. The cells were disrupted by sonication (Brautaset *et al*., 2003). The cell debris was removed by centrifugation (14.000 × g, 60 min, 4°C), and the supernatant was used as crude extract for measuring the lysine decarboxylase activity. Lysine decarboxylase activity was calculated by measuring the conversion of lysine to cadaverine over time using HPLC as described elsewhere (Kind *et al*., 2010). The assays were carried out at 50°C, and one unit of lysine decarboxylase activity was defined as the amount of enzyme that formed 1 μmol of cadaverine per min at 50°C. Protein concentration was determined using the assay of Bradford (Bradford, 1976).

### Cadaverine and L-lysine shake flask production studies

Production experiments were performed in 500 ml baffled shake flasks (Belco) containing 100 ml MeOH_200_ medium (Jakobsen *et al*., 2006). *B. methanolicus* strains were cultivated in triplicate cultures using inoculum made from exponentially growing cells (Brautaset *et al*., 2010; Nærdal *et al*., 2011). Samples for amino acid measurements were collected during the late exponential and stationary growth phases as described previously (Jakobsen *et al*., 2009; Nærdal *et al*., 2011), and measurements of cadaverine and amino acids were performed by using 9-fluorenylmethyl chloroformate (FMOC) or o-phthaldialdehyde derivatization and reverse-phase high-performance liquid chromatography (Jakobsen *et al*., 2009; Brautaset *et al*., 2010; Schneider and Wendisch, 2010). Concentrations for cadaverine are reported for the free base (MW of 102.18 g mol^−1^).

### High-cell-density fed-batch methanol fermentation

Fed-batch fermentation was performed at 50°C in UMN1 medium in Applikon 3-l fermentors with an initial volume of 0.75 litre essentially as described previously (Jakobsen *et al*., 2009; Brautaset *et al*., 2010). Chloramphenicol (5 μg ml^−1^) was added to the initial batch growth medium, the pH was maintained at 6.5 by automatic addition of 12.5% (wt/vol) NH_3_ solution, and the dissolved oxygen level was maintained at 30% saturation by increasing the agitation speed and using enriched air (up to 60% O_2_). The methanol concentration in the fermentor was monitored by online analysis of the headspace gas with a mass spectrometer (Balzers Omnistar GSD 300 02). The headspace gas was transferred from the fermentors to the mass spectrometer in insulated heated (60°C) stainless steel tubing. The methanol concentration in the medium was maintained at a set point of 150 mM by automatic addition of methanol feed solution containing methanol, trace metals and antifoam 204 (Sigma), as described in (Brautaset *et al*., 2010). The inoculum preparation protocol, the fermentation conditions and fermentation progress was as described previously (Brautaset *et al*., 2010). All fermentations were run until the carbon dioxide content of the exhaust gas was close to zero (no cell respiration). Bacterial growth was monitored by measuring the optical density at 600 nm (OD_600_). Dry cell weight was calculated using a conversion factor of one OD_600_ unit corresponding to 0.24 g dry cell weight per litre (calculated based on multiple measurements of dry cell weight and OD_600_ during the fermentation trial). Due to the significant the increase in culture volume throughout the fermentation, the biomass, cadaverine and amino acid concentrations were corrected for the increase in volume and subsequent dilution. The volume correction factor of 1.8 was used for values presented in Table 4. The actual concentrations measured in the bioreactors were therefore accordingly lower as described previously (Jakobsen *et al*., 2009). Samples for determination of volumetric cadaverine and amino acid yields were collected from early exponential phase and throughout the cultivation (10–47 h) and analysed as described above.
