# Methanol‐based cadaverine production by genetically engineered *Bacillus methanolicus* strains

**DOI:** 10.1111/1751-7915.13352

**Published:** 2018-12-21

## Specification of the mistake identified

The cadaverine concentrations reported were miscalculated and overestimated by 41.6% because the HPLC standard solution was made of cadaverine dihydrochloride (175.1 g/mol) not cadaverine (102.18 g/mol).

## Implications of the corrections

The identified mistake does not destroy the novelty of the paper and all main hypotheses and conclusions remain valid.

Minor adjustments due to the correction (changes in colour red)

1. The published sentence ‘*Notably, combined formation of cadaverine and L‐lysine by the cadA and ldcC expressing strains was above 3‐fold higher than L‐lysine formation by the parent strain (Table* 1 *), which might indicate feedback deregulation by L‐lysine as consequence of a metabolic pull by lysine decarboxylase*.’
  **Table 1:** Specific L‐lysine decarboxylase activities, cadaverine and L‐lysine production levels in recombinant *B. methanolicus* M168‐20 strains

Plasmid	L‐lysine decarboxylase specific activity	Cadaverine	L‐lysine	Cadaverine + L‐lysine
	nmol/min/mg protein	mg/L	mg/L	mg/L
pHP13	< 1 ± 0.2	0.0	140 ± 10	140
pTH1mp‐*ldcC*	7.0 ± 1.0	75 ± 6	40 ± 5	115
pTH1mp‐*cadA*	88.0 ± 11.0	245 ± 15	10 ± 2	255

The results shown are from triplicate (cadaverine and L‐lysine) and duplicate (lysine decarboxylase activity) shake flask cultures. Activity was measured using crude extracts from exponentially growing cells, whereas the production levels were found from late stationary cultures, approximately 20 h after inoculation.
  **Table 2:** Production of cadaverine and L‐lysine by recombinant *B. methanolicus* M168‐20 strains cultivated at different medium pH

pH	M168‐20(pHP13)		M168‐20(pTH1mp‐*ldcC*)		M168‐20(pTH1mp‐*cadA*)	
	Cadaverine	L‐lysine	Cadaverine	L‐lysine	Cadaverine	L‐lysine
6.5	0	50 ± 10	30 ± 3	< 15	26 ± 3	< 15
7.2	0	130 ± 10	78 ± 6	40 ± 5	250 ± 12	< 30
7.6	0	140 ± 10	185 ± 12	< 30	263 ± 12	< 30
8.0	0	140 ± 10	178 ± 18	< 15	292 ± 18	< 30
8.5	0	140 ± 10	178 ± 18	< 15	303 ± 18	< 30

The mean values (mg/L) and standard deviation of triplicate shake flask cultures are presented. The production levels were found from late stationary cultures, from 20‐30 h after inoculation.

should be adjusted to

‘*Notably, combined formation of cadaverine and L‐lysine by the cadA expressing strain was nearly 2‐fold higher than L‐lysine formation by the parent strain (Table* 1 *), which might indicate feedback deregulation by L‐lysine as consequence of a metabolic pull by lysine decarboxylase*’.

- The statement below concerning three different lysine decarboxylase expressing strains is no longer true for MGA3(pTH1mp‐*ldcC‐lysA*) because it in fact produces less cadaverine than strain MGA3(pTH1mp‐*lysA*) produces L‐lysine.

‘*Interestingly, L‐lysine production was in each case lower (7, 55 and 150 mg/L, respectively; Table* 3 *) for the three isogenic strains that do not express ldcC, i.e. MGA3(pHP13), MGA3(pTH1mp‐lysC) and MGA3(pTH1mp‐lysA), respectively, indicating that LdcC exerts a metabolic pull deregulating flux through the L‐lysine biosynthesis pathway*’.

**Table 3:** Cadaverine and L‐lysine production by recombinant *B. methanolicus* MGA3 strains

Plasmid	Cadaverine	L‐lysine
	mg/L	mg/L
pHP13	0	7 ± 1^a^
pTH1mp‐*lysC*	0	55 ± 5^a^
pTH1mp‐*lysA*	0	150 ± 10^a^
pTH1mp‐*ldcC*	12 ± 2	7 ± 1
pTH1mp‐*ldcC*‐*lysC*	82 ± 6	< 10
pTH1mp‐*ldcC*‐*lysA*	111 ± 6	< 10
pTH1mp‐*cadA*	262 ± 18	< 10
pTH1mp‐*cadA*‐*lysA*	280 ± 18	< 10

The production levels were found from late stationary shake flask cultures, approximately 20 h after inoculation.

**a**. Data imported from (Nærdal, I.*, et al*., 2011).

**Table 4:** Fed‐batch methanol fermentation production data of strains MGA3(pTH1mp‐*cadA*) and MGA3(pHP13)

Strain	CDW	μ^a^	Asp^b^	Glu^b^	Ala^b^	Lys^b^	Cad^b^
	g/L	h^−1^	g/L	g/L	g/L	g/L	g/L
MGA3(pTH1mp‐*cadA*)	65.5	0.45	1.5	71.8	10.2	0.0	6.5
MGA3(pHP13)	45.0	0.49	1.1	59.0	12.0	0.4	0.0

μ, specific growth rate; Ala, L‐alanine; Asp, L‐aspartate; Cad, Cadaverine; CDW, cell dry weight; Glu, L‐glutamate; Lys, L‐lysine.

The maximum mean values from early (CDW) or late stationary growth phase is presented for the MGA3(pTH1mp‐*cadA*) duplicate cultures and the deviation never exceed ten per cent. The MGA3(pHP13) data were imported from (Brautaset, T.*, et al*., 2010).

**a**. Specific growth rates are maximum values calculated from the exponential growth period.

**b**. CDW, cadaverine and amino acid concentrations are maximum values and volume corrected (see “Experimental Procedures” section).

## Summary

Methanol is regarded as an attractive substrate for biotechnological production of value‐added bulk products, such as amino acids and polyamines. In the present study, the methylotrophic and thermophilic bacterium *Bacillus methanolicus* was engineered into a microbial cell factory for the production of the platform chemical 1,5‐diaminopentane (cadaverine) from methanol. This was achieved by the heterologous expression of the *Escherichia coli* genes *cadA* and *ldcC* encoding two different lysine decarboxylase enzymes and by increasing the overall L‐lysine production levels in this host. Both CadA and LdcC were functional in *B. methanolicus* cultivated at 50°C and expression of *cadA* resulted in cadaverine production levels up to 300 mg l^−1^ during shake flask conditions. A volume‐corrected concentration of 6.5 g l^−1^ of cadaverine was obtained by high‐cell‐density fed‐batch methanol fermentation. Our results demonstrated that efficient conversion of L‐lysine into cadaverine presumably has severe effects on feedback regulation of the L‐lysine biosynthetic pathway in *B. methanolicus*. By also investigating the cadaverine tolerance level, *B. methanolicus* proved to be an exciting alternative host and comparable to the well‐known bacterial hosts *E. coli* and *Corynebacterium glutamicum*. This study represents the first demonstration of microbial production of cadaverine from methanol.

The authors apologize to all readers for the inconvenience caused to the identified mistake.
